# Overexpression of MAP2 and NF-H Associated with Dendritic Pathology in the Spinal Cord of Mice Infected with Rabies Virus

**DOI:** 10.3390/v10030112

**Published:** 2018-03-06

**Authors:** Jeison Monroy-Gómez, Gerardo Santamaría, Orlando Torres-Fernández

**Affiliations:** 1 Grupo de Morfología Celular, Instituto Nacional de Salud (INS), Bogotá 111321, D.C., Colombia; jmonroy@ecr.edu.co or jeison-monroy@hotmail.com (J.M.-G.); biojulmilger@gmail.com (G.S.); 2 Departamento de Ciencias Básicas, Institución Universitaria Escuela Colombiana de Rehabilitación, Bogotá 110121, D.C., Colombia

**Keywords:** rabies virus, spinal cord, MAP2, NF-H, dendrite pathology, immunohistochemistry, Golgi–Cox

## Abstract

Rabies is a viral infection that targets the nervous system, specifically neurons. The clinical manifestations of the disease are dramatic and their outcome fatal; paradoxically, conventional histopathological descriptions reveal only subtle changes in the affected nervous tissue. Some researchers have considered that the pathophysiology of rabies is based more on biochemical changes than on structural alterations, as is the case with some psychiatric diseases. However, we believe that it has been necessary to resort to other methods that allow us to analyze the effect of the infection on neurons. The Golgi technique is the gold standard for studying the morphology of all the components of a neuron and the cytoskeletal proteins are the structural support of dendrites and axons. We have previously shown, in the mouse cerebral cortex and now with this work in spinal cord, that rabies virus generates remarkable alterations in the morphological pattern of the neurons and that this effect is associated with the increase in the expression of two cytoskeletal proteins (MAP2 and NF-H). It is necessary to deepen the investigation of the pathogenesis of rabies in order to find therapeutic alternatives to a disease to which the World Health Organization classifies as a neglected disease.

## 1. Introduction

The cytoskeleton helps maintain the form and internal organization of cells. Additionally, it provides mechanical support that allows cells to perform essential functions such as division and movement. It is composed of three structural complexes: microtubules (MT), neurofilaments (NF), and microfilaments (MF) [1]. MTs are composed of α- and β-tubulin dimers to which specific proteins called microtubule-associated proteins (MAPs) are attached [2]. Isoforms from the MAP2 family (MAP2A and MAP2B) are expressed exclusively in mature neurons; they are involved in the stabilization and strengthening of the rigidity of microtubules, inhibition of polymerization, and modulation of neurite development [3]. NFs are the most rigid and resilient elements of the cytoskeleton. NF-H (heavy) is expressed when radial axon growth is required for development of the nervous system [4]. Its function is fundamentally structural; it is involved in radial axonal growth during development, maintaining axonal caliber and the speed of nerve conduction [5].

Rabies is an illness caused by a virus that primarily affects the central nervous system. The virus almost exclusively affects neurons, resulting in notable neurological changes without any apparent morphological changes in the affected nervous tissue [6]. Even neuronal death from apoptosis is not considered important in the pathology. Instead, the effects of rabies are attributed to biochemical alterations [7,8]. 

In addition to its exclusive neurotropic feature, rabies differs from other viruses because it does not induce viremia and has the highest mortality of any known infectious disease (more than Ebola, for example) [6]. Another characteristic of rabies is its apparently late effect on different pathophysiological parameters; the electroencephalogram pattern is preserved unchanged for almost the entire duration of the disease and collapses only a few hours before the disease in both humans and animal models. Likewise, antibody titers are very low during the development of human rabies and only reach high levels a few hours before the death of the patient. This means that the neurons survive the infection and maintain their complex functional mechanisms late into the disease [9]. Perhaps the late manifestations of rabies are due to the fact that the virus allows the preservation of the integrity of the neural network until well-advanced infection as a survival strategy and to facilitate its transmission [10]. These characteristics of rabies motivate to direct the investigation of their pathogenesis from a neuroscientific approach.

Currently, little is known about the effects of rabies viral infection on cytoskeletal proteins. Studies have shown changes in the immunoreactivity of MAP2 and NF-H [11,12] and loss of actin filaments [13]. Additionally, dendritic pathology occurs in the cerebral cortex of mice infected with rabies [14,15]. Generally, dendritic pathology is linked to the loss of MAP2 [16]. These results suggest that the degeneration of neuronal processes due to the disruption of cytoskeletal integrity from rabies viral infection is a component of neural dysfunction [6]. 

Nevertheless, most studies have focused more on the effects of the infection on the brain, even though the spinal cord is the first structure infected in the central nervous system [17]. Paralysis of the posterior extremities and quadriplegia generated by infection with rabies virus in both humans and animal models [18] demonstrate how the virus compromises the motor system and especially the spinal cord [19]. Consequently, the purpose of this study was to investigate the effect of infection with rabies virus on the expression of two cytoskeletal proteins (MAP2 and NF-H) and its link to dendritic pathology in neurons of the ventral horn of the mouse spinal cord.

## 2. Materials and Methods

### 2.1. Ethics Statement

This research on rabies virus in mice (Project Code Colciencias 210465740573, Contract 639 of 2014) was discussed and approved among the staff members of the Ethics Committee of Colombia’s National Institute of Health (Instituto Nacional de Salud—INS). The protocol for use of animals was approved by them as well and all the procedures were carried out in accordance with the approved guidelines (with meeting record No. 4-2014 of 29 May 2014).

### 2.2. Animal Culture Handling 

Biological material was obtained from 4-week-old (±28 days) Institute of Cancer Research (ICR) strain female mice with an average weight of 19 g. Animals were randomly selected for group placement. The mice were confined to a high-security room in the vivarium of INS under environmental and nutritional conditions in accordance with national (Colombian Law 84 of 1989) and international ethics regulations required for laboratory animal studies. The mouse is the most widely used animal model for research and diagnosis of rabies.

### 2.3. Viral Infection, Fixing, and Extracting of the Spinal Cord

Viral infection was carried out with aliquots of the Challenge Virus Standard (CVS) strain of rabies virus obtained from the Virology Laboratory of the INS. Ten mice were injected intramuscularly in the hamstring muscle of the hind left leg with 0.03 mL of rabies virus equivalent to 33,800 Median lethal dose (DL50), while the control group (10 mice) was injected with a diluted solution lacking the virus (2% normal equine serum in distilled water and antibiotics (200 IU/mL penicillin and 4 mg/mL streptomycin)). Animals were observed over the following days until they were sacrificed (when in the terminal state or lying prostrate on the bottom of the cage). Spinal cord tissue was obtained after anesthetizing the mice with 0.2 mL 30% chloral hydrate (Merck, Darmstadt, Germany) injected intraperitoneally. Once the animals were unconscious, tissues in five infected and five control animals were fixed through transcardial perfusion; approximately 30 mL of 7.3 pH phosphate buffer solution (PBS) was circulated for 3 min, followed by the fixing solution of approximately 70 mL of 4% paraformaldehyde (PFA). Once the fixative had run through the perfusion, PBS was circulated for an additional minute. The spinal cord was carefully removed and transferred to a fresh solution of 4% PFA. The animals used for Golgi–Cox staining (the remaining five infected and five control animals) were perfused with saline solution [12,14,17]. 

### 2.4. Immunohistochemistry

Cervical cord sections were embedded in 4% agar, then mounted onto a vibratome (VT1000-S, Leica Biosystems Nussloch GmbH, Heidelberger, Germany), producing a series of cross-sections 40 micrometers thick. The sections were placed in 2.5 cm diameter Petri dishes containing PBS and stored at 20–22 °C and 30% humidity. The floating sections were kept under constant agitation using a 90 rpm agitator/vibrator. 

An indirect immunohistochemistry method, specifically the avidin–biotin complex staining method, was used to detect antigens for MAP2, NF-H, and rabies virus. Cervical cord slices were washed in PBS and agitated overnight. They were then incubated in NH_4_Cl (Merck) to remove aldehyde residuals and treated with H_2_O_2_ (Merck) to inhibit endogenous peroxidase activity. Non-specific antigens were blocked with a solution of normal horse serum (Merck Millipore, Darmstadt, Germany), bovine serum albumin (Sigma, St. Louis, MO, USA), and Triton X-100 (MP-Biomedicals, LLC, Fountain Parkway, USA). 

Antigens of interest were evaluated by incubating slices with the following antibodies. Rabies virus was detected using polyclonal anti-rabies antiserum (1:2500 dilution) obtained in rabbit [20]. The effect of the virus on the cytoskeleton was evaluated using polyclonal anti-MAP2 (Santa Cruz Biotechnology, Inc., Dallas, TX, USA) (1:2500 dilution) and monoclonal anti-NF-200 (Sigma) (1:1000 dilution). Slices processed without the primary antibodies were used as negative controls.

Slices underwent secondary antibody incubation with anti-rabbit (Sigma) (1:600 dilution) for MAP2 and rabies immunohistochemistry and with anti-mouse (Sigma) (1:600 dilution) for NF-H. The slices were later treated with avidin–biotin complex (ABC) (Ref. SK4000, Vector Laboratory INC, Burlingame, CA, USA). Immunostaining was performed using a diaminobenzidine-nickel kit (DAB-Nickel, Ref. PK4100, Vector Laboratory INC). The slices were then laid out on glass slides pretreated with gelatin (Merck), allowed to dry, and mounted with Entellan^®^. 

### 2.5. Golgi–Cox Method 

The modified Golgi–Cox method was used to study dendritic pathology [21]. Sections of the spinal cord of saline-perfused animals were soaked in a Cox solution (5% potassium dichromate, 5% mercury chloride, and 5% potassium chromate), prepared three months in advance, for 14 days. The sections were later transferred to a 30% sucrose solution for 5 to 10 days. Two-hundred-micrometer cross-sections were obtained using the Leica vibratome and stored in 6% sucrose. The slices were washed briefly in distilled water, treated with ammonium hydroxide for 30 min, washed again, and transferred to a Kodak photographic fixer (in darkness for 30 min). Finally, sections were dehydrated in ethanol and xylene and mounted in undiluted Canada balsam (Sigma) [14,15].

### 2.6. Quantitative and Qualitative Analysis 

Qualitative observations of immunostaining were performed to detect the presence and location of immunoreactive cells in response to each of the antigens evaluated under normal conditions and in samples from infected animals. Five infected animals (experimental units) and their respective controls were used for the quantitative analysis. Five slices (repetitions) were selected for each experimental unit. Images were digitized with CAPTURE PRO 6, and optic densitometry analysis of the immunohistochemistry reactions was performed using free ImageJ (NIH) software. This program has a system that allows obtaining an average of optical density according to a gray scale in which light absorbance levels of the cuts are measured. The resulting data were analyzed with the statistical program Infostat (version 2017, Infostat Inc., Cordoba, Argentina). Prior to this analysis, the normality of the data was compared using the Shapiro–Wilk test. Group comparison was performed using the paired Student’s *t*-test.

## 3. Results

### 3.1. Diagnosis of Rabies Virus Infection 

**Figure 1:**
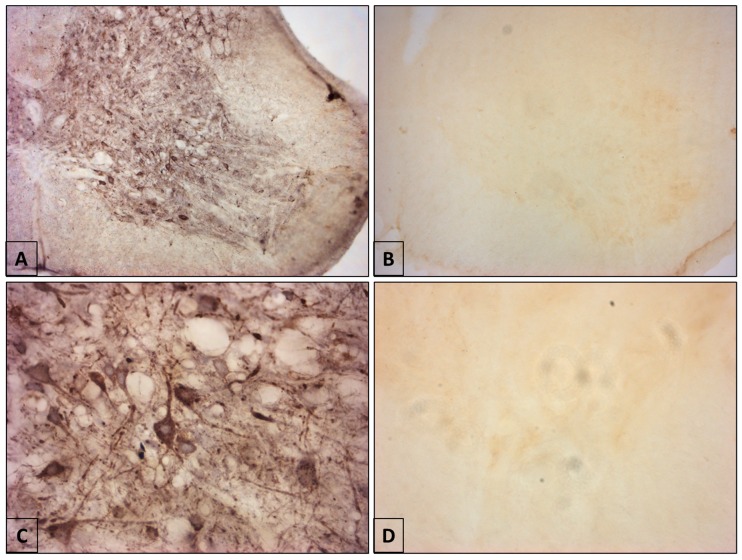
Rabies immunoreactivity in the spinal cord of mice. (**A**,**C**) Infected mouse. (**A**) Note the presence of rabies antigens in all the cells of the cervical spinal cord (10×). (**C**) Neurons in the ventral horn are marked as positive for rabies. The distribution of viral antigens throughout the cytoplasm illustrates the cellular morphology (40×). (**B**,**D**) Control mouse. Note non-immunoreactivity and non-labeling of viral antigens. (**B**) (10×) and (**D**) (40×).

Rabies virus infected 100% of the inoculated mice. The infected animals showed symptoms from the third day post-inoculation, characterized by pulling back one or both forelegs, ruffled fur, and weight loss with respect to the control animals. Prostration, defined by total loss of mobility in the posterior extremities, drastic weight loss, and cold body temperatures on contact, was observed between the fifth and sixth day post-inoculation. The anti-rabies antibody can detect the presence of viral antigens throughout the gray matter of the ventral horn of the spinal cord. Rabies immunoreactivity was confirmed through the presence of immunostained antigens in the cytoplasm of the soma and neuronal processes of the ventral horn of the spinal cord (Figure 1A,B).

### 3.2. Immunoreactivity to Microtubule-Associated Protein 2 and Heavy Neurofilamets and the Effect of Rabies Virus Infection 

**Figure 2:**
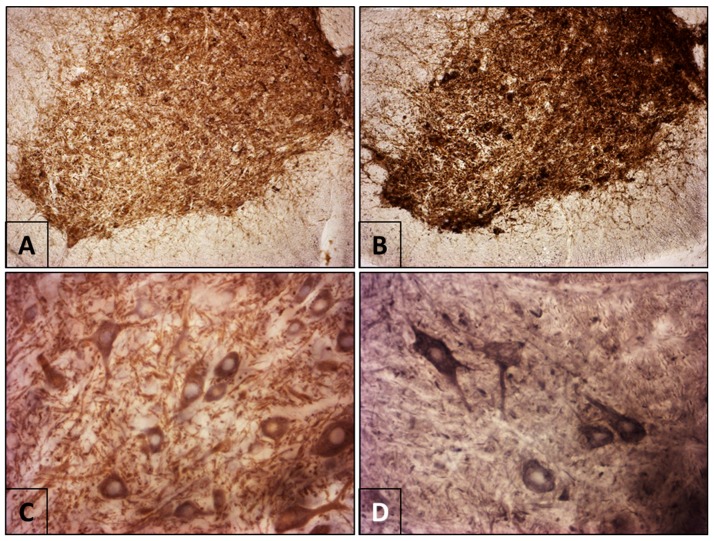
Immunoreactivity of Microtubule-Associated Protein (MAP2) in the mouse spinal cord. (**A**,**C**) Control mouse. (**A**) There is notable immunoreactivity of the soma and neuropil in all of the laminae of the ventral horn. (**C**) At higher magnification, heavy staining is evident in the cytoplasm (perikaryon) but not in the nuclei. (**B**,**D**) Infected mouse. Infection with rabies virus increased immunoreactivity to MAP2 in the neurons and neuropil of the spinal cord (Panoramic images (**A**,**B**) are at 10× magnification. (**C**,**D**) are at 40×).

**Figure 3:**
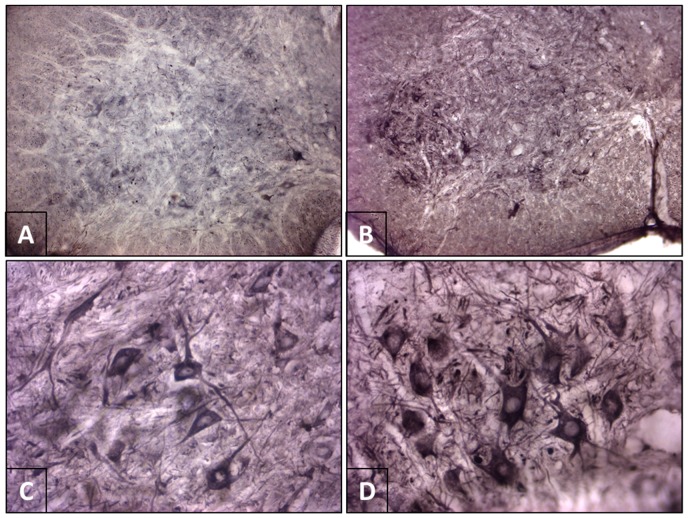
Immunoreactivity to heavy neurofilamets (NF-H) in the mouse spinal cord. (**A**,**C**) Control mouse. (**A**) The panoramic image reveals diffuse staining in the gray matter. (**B**) At higher magnification, notable immunostaining is evident in the perikaryon (but not the nucleus) and the neural processes. (**B**,**D**) Infected mouse. Infection with rabies virus resulted in increased immunoreactivity to NF-H in neurons and neuropil in the spinal cord. Note the greater number of processes in the infected animal (**D**) in comparison to the control (**C**) (Panoramic images (**A**,**B**) are at 10× magnification. (**C**,**D**) are at 40×).

Samples from the control animals showed immunoreactivity to MAP2 distributed throughout the gray matter of the spinal cord and in neurons of different sizes. The amount of staining allowed for a detailed description of the morphology of the cell bodies and some of their processes. Immunostaining was observed in the cytoplasm but not within the nucleus of the neurons (Figure 2A,C). Immunoreactivity to NF-H in the controls exhibited staining of a similar pattern but less intense than that seen for MAP2 (Figure 3A,C). 

Rabies infection resulted in increased immunoreactivity of both MAP2 (Figure 2B,D) and NF-H (Figure 3B,D), with a more intense staining in the soma and neural processes found in both the gray and white matter. The increase in immunostaining for MAP2 and NF-H was most evident in the neurons of lamina IX. Optical densitometry of the comparison between the control and infected samples demonstrated a statistically significant increase in the immunoreactivity of MAP2 (*p* = 0.0135) and NF-H (*p* = 0.0331) in the infected samples (Table 1). 

Each datum corresponds to the average value from five slices, with smaller values indicating less light transmitted (greater optical density) and therefore an increase in protein concentration. The scale for light transmission is 0–255, where 0 is black and 255 is white.

### 3.3. Dendritic Pathology in Spinal Cord Neurons from Mice Infected with Rabies

**Figure 4:**
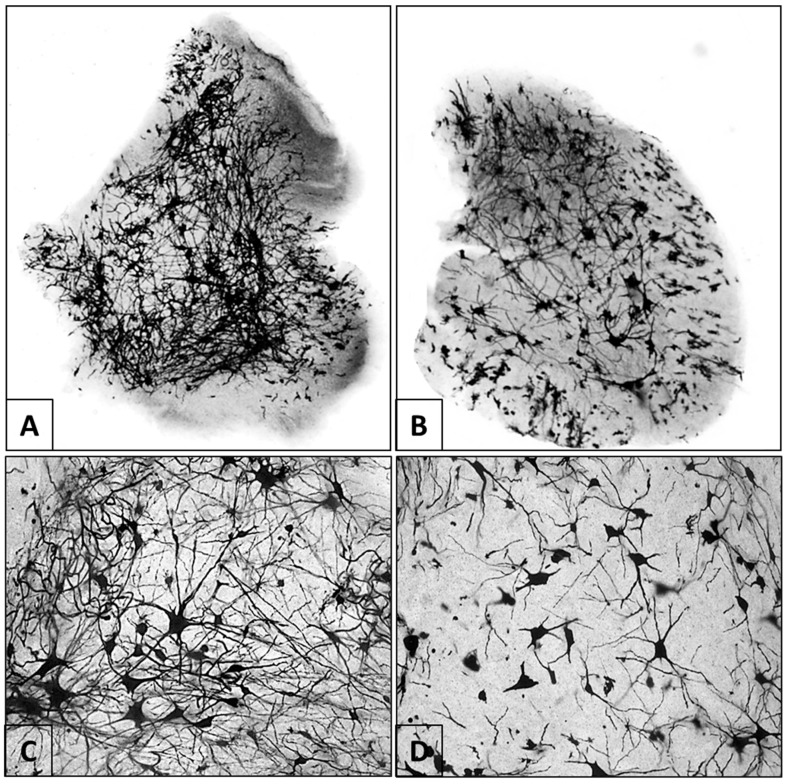
Golgi–Cox staining in samples from the control and infected groups. (**A**,**C**) Control mouse. Soma and a dense dendritic net are observed in the ventral horn of the spinal cord. Panel **C** highlights a motor neuron in the center of the image. (**B**,**D**) Infected mouse. Infection with rabies virus resulted in a notable decrease in the number and length of the dendrites of each of the neurons. This is reflected in a lower-density dendritic net. (Panoramic images (**A**,**B**) are at 5× magnification. (**C**,**D**) are at 40×).

The Golgi technique was used to describe the morphology of neurons found in gray matter. This included the soma or neural body and numerous intertwined dendrites, forming a dense net. Motor neurons were notable for their larger size and star-like appearance, which confer diameters equal to the dendrites projecting from their soma. The average diameter of the somata was similar between the control and the infected animals (*n* = 25; control: *X* = 23.81 µm; range: 10.5–39.2 µm; infected: *X* = 21.69 µm; range: 11–35.2 µm). However, some alterations were evident in the morphology of the dendritic pattern of the infected cells, specifically, fewer branches and shorter dendrite length. The most evident deviation was a notably less dense dendritic net in the samples from the animals infected with rabies virus (Figure 4).

## 4. Discussion

The function of neurofilaments is to provide mechanical resistance to the cell as well as regulate the distribution of proteins and the transmission of intracellular signals. The C-terminal domain of NF-H form fine lateral extension that increases the spacing between NFs, thus maximizing their ability to occupy space during axon caliber expansion and the propagation of action potentials down the length of the axon [5]. MAP2 is responsible for maintaining the stability and increasing the rigidity of microtubules as well as inhibiting polymerization and modulating neurite generation. They are also involved in the formation of cross-links between microtubules and neurofilaments [3]. 

In this study, infection with rabies virus resulted in increased immunoreactivity of MAP2 and NF-H in the spinal cord of mice. The expression of MAP2 in the cerebral cortex of mice increases following injection with the two types (fixed and street) of rabies virus [12], and MAP2 is overexpressed in patients with encephalic rabies [22]. Rabies virus also affects the expression of other proteins. An increase in parvalbumin (PV), a calcium-binding protein, has been shown in the cerebral cortex [23], the cerebellum [24], and the spinal cord of mice infected with rabies [17]. Prosniak et al. showed that the expression of at least 16 genes (1.4% of the genes evaluated in the study) increased in the brains of mice inoculated with CVS rabies virus [25]. In contrast, Song et al. did not show any qualitative variation in the immunoreactivity of MAP2 in the pyramidal neurons of the hippocampus of infected mice [13]. Other studies have shown diminished immunoreactivity of MAP2 and NF-H in neurons infected in vitro [11], as well as loss of expression of MAP2 in neurons of the cerebral cortex and cerebellar peduncle [26] and loss of actin filaments in the hippocampus of mice [13] and in cell cultures [27]. It is possible that these differences are due to the type and virulence of the viral strains used in the studies [26]. However, proteomic analysis of the proteins in the brains of humans infected with rabies [22] supports the increased expression of MAP2 and NF-H reported here.

Overexpression of MAP2 and NF-H could be a mechanism to facilitate dissemination of the virus. It has been suggested that the cytoskeleton plays a fundamental role in the retrograde transport of the rabies virus [28,29], so it is possible that the virus has the capacity to be actively transported through the microtubules, capable of interacting with the cytoskeletal motor proteins of the dynein family [30,31].

One of the mechanisms that could facilitate retrograde rabies virus transport is binding to p75NTR receptors, which internalizes the virus via the p75 pathway. This increases transport rates by activating different signaling pathways, resulting in the binding of various ligands or interactions with diverse co-receptors that up-regulate retrograde axonal transport [32]. It is possible that the binding of rabies virus to p75NTR clusters in the cell membrane promotes the recruitment and mobilization of both microtubules and dynein [32]. Additionally, the P viral protein of rabies virus is linked to the 10-kDa dynein light chains (LC8). This protein is involved in the movement of negative organelles down the length of the microtubules [33]. Similarly, the rabies viral polymerase L forms a binding complex with dynein light chain 1 (DLC1), which is involved in the linking, modification, and post-translational modification of microtubules and in the regulation of the primary transcription of rabies virus [34]. This demonstrates that the dynein can be involved in the axonal transport of rabies virus along the length of the microtubules through the neuronal cells [33]. 

A link has been demonstrated between DLC1 and the MAP proteins in the direct binding to tubulin dimers [35]. This finding suggests that the virus not only uses the microtubule network during entry but also actively manipulates the cytoskeleton and associated proteins to achieve rapid and efficient transport [34]. It is possible that overexpression of MAP2 and NF-H is induced by DLC1 or by expression of a viral protein, as occurs in the post-translational modification associated with DLC1-polymerase L [34], DLC8, and P protein [33], and in the transport mediated by p75NTR [32]. These steps may constitute a set of mechanisms that together facilitate and accelerate viral transport. The HIV-1 virus also induces microtubule formation to facilitate infection [36]. 

Loss of MAP2 expression is generally linked to alterations in dendritic morphology [16]. However, our results seem to show that in rabies infection, dendritic pathology is linked to increased MAP2 expression. The Golgi–Cox method revealed images that showed morphological alterations in neurons of the ventral horn of the spinal cord, specifically a smaller number of dendrites that were shorter in length. Similar results were shown in the pyramidal neurons of the cerebral cortex of mice [12,14,15]. A link between increased MAP2 expression and dendritic pathology in Down’s syndrome has also been established [37]. The interruption of the cytoskeleton by microtubule depolymerization is due to loss of α-tubulin caused by infection with rabies virus via the formation of an M-protein filament network [27], which confirms that rabies infection damages the cytoskeleton of the host cell and is associated with the accumulation of viral M proteins.

Another possible explanation for this phenomenon could be linked to the accumulation of NF. Small increases in the expression of NF-H generate a large amount of total neurofilament and larger myelinated axons, while larger increases in NF-H result in decreased total neurofilaments and strongly inhibit their growth in transgenic mice [38]. Overexpression of NF-H results in anomalies in the organization and formation of neurofilaments that can cause alterations in cell morphology and metabolism [39]. The spinal cords of transgenic mice overexpressing NF-H and NF-M (medium) showed that an increase in one or both proteins causes an aggregation of NF and a separation of the NF network and microtubules, resulting in a drastic reduction of dendritic arborization in motor neurons [40]. Dendritic pathology in rabies has also been observed in the ultrastructural features of the distal dendrites of pyramidal neurons in the cerebral cortex, where there is evidence that the virus alters the organization of microtubules and induces appearance of myelin-like substances [41]. 

It is possible that, at the initial stages of the rabies infection, the increases in MAP2 and NF-H favor the speed of viral transport, but at later stages these continuing increases promote alterations in the cytoskeleton, which are then reflected in the cell morphology. These changes could be due to accumulation of NF-H in neurons, especially in motor neurons, impeding the interaction between NF and microtubules [40,42].

The behavioral and motor changes associated with rabies virus and the results reported in this research indicate that rabies provides a possible model for the study of neurodegenerative diseases. This is due to the association of the accumulation of the cytoskeleton proteins with the pathophysiology of neurodegenerative diseases. Recently, the neural intermediate filament inclusion disease (NIFID) and its association with an early onset frontotemporal dementia variant has been reported [43] and it has been shown that increases in heavy phosphorylated neurofilament (pNF-H) generate a higher rate of progression of amyotrophic lateral sclerosis [44]. Additionally, it has been suggested that elevated levels of neurofilaments in the spinal fluid are associated with dementias affecting the subcortical brain regions and with frontotemporal dementia [45].

In summary, this study confirmed the possible link between overexpression of MAP2 and NF-H and dendritic pathology in rabies. Nevertheless, it is necessary to continue investigating the involvement of neural receptors, the post-transductional regulation of the cytoskeleton in the transport of rabies virus, and the virus’s relationship with dendritic pathology, with the aim of understanding the mechanisms of neuronal dysfunction and generate in the future possible treatments to cure rabies.

## Figures and Tables

**Table 1:** Optic densitometry of the immunoreactivity to MAP2 and NF-H in control and infected samples.

	MAP2			NF-H		
Sample	Control	Infected	Difference	Control	Infected	Difference
1	79.87	58.57	21.30	119.11	89.82	29.18
2	100.65	74.39	26.26	122.00	104.37	15.63
3	84.50	83.04	1.46	86.46	82.11	4.35
4	81.33	60.72	20.61	100.61	93.75	6.86
5	99.39	78.52	20.87	80.09	47.86	32.23
Mean	89.15 ± 10	71.05 ± 10	18.10 ± 9.5	101.65 ± 8	83.58 ± 21	18.07 ± 12
*p*-Value		0.0135 **			0.0331 **	

** Difference is statistically significant (paired *t*-test).
